# Development of SyneBrick Vectors As a Synthetic Biology Platform for Gene Expression in *Synechococcus elongatus* PCC 7942

**DOI:** 10.3389/fpls.2017.00293

**Published:** 2017-03-02

**Authors:** Wook Jin Kim, Sun-Mi Lee, Youngsoon Um, Sang Jun Sim, Han Min Woo

**Affiliations:** ^1^Clean Energy Research Center, Korea Institute of Science and TechnologySeoul, South Korea; ^2^Green School (Graduate School of Energy and Environment), Korea UniversitySeoul, South Korea; ^3^Department of Chemical and Biological Engineering, Korea UniversitySeoul, South Korea; ^4^Department of Food Science and Biotechnology, Sungkyunkwan UniversitySuwon, South Korea

**Keywords:** *Synechococcus elongatus* PCC 7942, SyneBrick vectors, synthetic biology, gene expression, T7 promoters

## Abstract

Cyanobacteria are oxygenic photosynthetic prokaryotes that are able to assimilate CO_2_ using solar energy and water. Metabolic engineering of cyanobacteria has suggested the possibility of direct CO_2_ conversion to value-added chemicals. However, engineering of cyanobacteria has been limited due to the lack of various genetic tools for expression and control of multiple genes to reconstruct metabolic pathways for biochemicals from CO_2_. Thus, we developed SyneBrick vectors as a synthetic biology platform for gene expression in *Synechococcus elongatus* PCC 7942 as a model cyanobacterium. The SyneBrick chromosomal integration vectors provide three inducible expression systems to control gene expression and three neutral sites for chromosomal integrations. Using a SyneBrick vector, LacI-regulated gene expression led to 24-fold induction of the eYFP reporter gene with 1 mM isopropyl β-D-1-thiogalactopyranoside (IPTG) inducer in *S. elongatus* PCC 7942 under 5% (v/v) CO_2_. TetR-regulated gene expression led to 19-fold induction of the GFP gene when 100 nM anhydrotetracycline (aTc) inducer was used. Gene expression decreased after 48 h due to degradation of aTc under light. T7 RNA polymerase-based gene expression resulted in efficient expression with a lower IPTG concentration than a previously developed pTrc promoter. A library of T7 promoters can be used for tunable gene expression. In summary, SyneBrick vectors were developed as a synthetic biology platform for gene expression in *S. elongatus* PCC 7942. These results will accelerate metabolic engineering of biosolar cell factories through expressing and controlling multiple genes of interest.

## Introduction

Increasing concerns about limited fossil fuels and the advent of global warming have drawn attention to direct conversion of CO_2_ to high-value products through engineering cyanobacteria and microalgae (Ducat et al., 2011; Angermayr et al., 2015). These methods have high productivity per acre compared to terrestrial crops (Parmar et al., 2011). Metabolic engineering of cyanobacteria has been applied to development of biosolar factories from CO_2_ with the aim of enhancing production of bio-products through modification of metabolism and introduction of heterologous metabolic pathways (Atsumi et al., 2009; Liu et al., 2011; Choi et al., 2016; Chwa et al., 2016; Lee et al., 2017).

To reconstruct heterologous metabolic pathways in a cyanobacterium, multiple genes must be inserted into either a plasmid or the genome. Controllable expression of those genes is required to balance metabolic flux toward desired products. Tools for controlling gene expression in cyanobacteria have been developed by engineering TetR-regulated promoters in *Synechocystis* sp. PCC 6803 (Huang and Lindblad, 2013). Other tools include isopropyl β-D-1-thiogalactopyranoside (IPTG) and anhydrotetracycline (aTc)-based inducible promoters and a transacting small RNA system for *Synechococcus* sp. PCC 7002 (Markley et al., 2015; Zess et al., 2016), designed oxygen-responsive genetic circuits in *Synechocystis* sp. PCC 6803 (Immethun et al., 2016), and theophylline riboswitches in *Synechococcus elongatus* PCC 7942 (Nakahira et al., 2013). In addition, synthetic biology-based plasmid vectors (e.g., pPMQAK1) have been developed for *Synechocystis* sp. PCC 6803 (Huang et al., 2010). A synthetic platform for gene expression in *S. elongatus* PCC 7942, a model cyanobacterium for metabolic engineering and circadian rhythms, has not been reported.

This study aimed to develop SyneBrick™ vectors as synthetic platform for controllable gene expression in *S. elongatus* PCC 7942 under 5% (v/v) CO_2_. The standardization of biological parts and their assembly is a core idea behind synthetic biology. Standardized assembly approaches such as BglBrick cloning do not require PCR amplification and use sequence-based homologous recombination. Thus, these cloning methods are not limited by automation or in the number of DNA fragment copies used (Hillson, 2011). BglBrick standard vectors and the derivative CoryneBrick vectors have been successfully applied for metabolic engineering of *Escherichia coli* and *Corynebacterium glutamicum* to express multiple target genes for synthetic biology (Lee et al., 2011, 2014; Kang et al., 2014). Based on these synthetic biology platforms, we developed SyneBrick vectors with regulatory and promoter sections for controlling gene expression. We expect that SyneBrick vectors will facilitate metabolic engineering applications in *S. elongatus* PCC 7942.

## Materials and methods

### Bacterial strains and growth conditions

Bacterial strains used in this work are in Table 1. For cloning, *E. coli* strains were grown in lysogeny broth medium (10 g/L tryptone, 5 g/L yeast extract, and 10 g/L NaCl) at 37°C. When appropriate, medium was supplemented with 25 μg/mL chloramphenicol, 50 μg/mL kanamycin, 100 μg/mL spectinomycin. *S. elongatus* PCC 7942 (wild type). Derivatives were cultivated at 30°C in 100 mL glass bottles under continuous fluorescent light (100 μmol photons/m^2^/s) in BG-11 medium (1.5 g/L NaNO_3_, 0.006 g/L ferric ammonium citrate, 0.001 g/L Na_2_EDTA·2H_2_O, 0.039 g/L K_2_PO_4_, 0.075 g/L MgSO_4_·7H_2_O, 0.020 g/L Na_2_CO_3_, 0.036 g/L CaCl_2_·2H_2_O, 0.006 g/L citric acid, 2.860 mg/L H_3_BO_3_, 1.810 mg/L MnCl_2_·4H_2_O, 0.222 mg/L ZnSO_4_, 0.39 mg/L Na_2_MoO_4_·2H_2_O, 0.079 mg/L CuSO_4_·5H_2_O, 0.0494 mg/L Co[NO_3_]_2_·6H_2_O) supplemented with 10 mM MOPS (pH 7.5). Supplied at a constant flow rate of 10 mL/min into medium was 5% (v/v) CO_2_ gas and 95% (v/v) air. Selection was with 10 μg/mL spectinomycin, 10 μg/mL kanamycin or 3 μg/mL chloramphenicol. Isopropyl-β-D-1-thiogalactopyranoside (IPTG) was added into culture medium at inoculation for induction. Cell growth was monitored by OD_730_ with a UV spectrophotometer (Agilent Technology, CA, USA).

**Table 1:** **Bacterial strains and plasmids used in this work**.

**Strains or plasmids**	**Relevant characteristics**	**Source or references**
**STRAINS**		
*E. coli* HIT-DH5α	F^−^(80d *lac*Z M15) (*lac*ZYA-*arg*F) U169 *hsd*R17(r^−^ m^+^) *rec*A1 *end*A1 *rel*A1 *deo*R96	Invitrogen
*E. coli* BL21(DE3)	F^−^*ompT gal dcm lon hsdS_*B*_*(*r_*B*_*^−^*m_*B*_*^−^) λ(DE3 [*lacI lacUV5*-*T7p07 ind1 sam7 nin5*]) [*malB*^+^]_*K*-12_(λ^S^)	Studier and Moffatt, 1986
*S. elongatus* PCC7942	Wild type (ATCC 33912)	ATCC
Se1Bb1s-GFP	*S. elongatus* PCC 7942 NSI::Bb1s-GFP	Chwa et al., 2016
Se1Bb1s-eYFP	*S. elongatus* PCC 7942 NSI::Bb1s-eYFP	This study
Se1Bb2s-GFP	*S. elongatus* PCC 7942 NSI::Bb2s-GFP	Chwa et al., 2016
Se1Bb1s-None	*S. elongatus* PCC 7942 NSI::Bb1s	This study
Se2Bb1k-eYFP	*S. elongatus* PCC 7942 NSII::Bb1s-eYFP	This study
Se2Bb7k-eYFP	*S. elongatus* PCC 7942 NSII::Bb7k-eYFP	This study
Se2Bb1k-None	*S. elongatus* PCC 7942 NSII::Bb1k	This study
Se3Bb1c-GFP	*S. elongatus* PCC 7942 NSIII::Bb1c-GFP	This study
Se23Bb7kc-eYFP/P	*S. elongatus* PCC 7942 NSII::Bb7k-eYFP NSIII::Bb1c-T7RNAP	This study
Se23Bb7.3kc-eYFP/P	*S. elongatus* PCC 7942 NSII::Bb7.3k-eYFP NSIII::Bb1c-T7RNAP	This study
Se23Bb7.4kc-eYFP/P	*S. elongatus* PCC 7942 NSII::Bb7.4k-eYFP NSIII::Bb1c-T7RNAP	This study
**PLASMIDS**		
pBbE1k-RFP	ColE1, km^r^, P*_*tetA*_, rfp*	Lee et al., 2011
pBbE2c-RFP	ColE1, Cm^r^, P*_*tetA*_, rfp*	Lee et al., 2011
p416-TEF-eYFP	pBR_322, Amp^r^, P_*t*3_,*eyfp (or yECitrine)*	Alper et al., 2006
pSe1Bb1s-GFP	pUC, Spc^r^, LacI, P*_*trc*_, gfp*, NSI target sites	Chwa et al., 2016
pSe1Bb1s-eYFP	pUC, Spc^r^, LacI, P*_*trc*_*, ey*fp*, NSI target sites	This study
pSe1Bb1s	pUC, Spc^r^, LacI, P*_*trc*_*, NSI target sites	This study
pSe1Bb2s-GFP	pUC, Spc^r^, TetR, P*_*tetA*_, gfp*, NSI target sites	This study
pSe2Bb1k-GFP	pUC, Km^r^, LacI, P*_*trc*_*, g*fp*, NSII target sites	Chwa et al., 2016
pSe2Bb1k-eYFP	pUC, Km^r^, LacI, P*_*trc*_*, ey*fp*, NSII target sites	This study
pSe2Bb1k	pUC, Km^r^, LacI, P*_*trc*_*, NSII target sites	This study
pSe2Bb7k-eYFP	pUC, Km^r^, P_**T*7*_, NSII target sites, ey*fp* T7 promoter (Original strength)	This study
pSe2Bb7.3k-eYFP	pUC, Km^r^, P_**T*7.3*_, NSII target sites, ey*fp* T7.3 promoter of Temme et al. (2012)	This study
pSe2Bb7.4k-eYFP	pUC, Km^r^, P_**T*7.4*_, NSII target sites, ey*fp* T7.4 promoter of Temme et al. (2012)	This study
pSe3Bb1c-GFP	pUC, Cm^r^, LacI, P*_*trc*_*, g*fp*, NSIII target sites	This study
pSe3Bb1c-T7RNAP	pUC, Cm^r^, LacI, P*_*trc*_*, NSIII target sites, *T7 RNAP* gene 1 from *E. coli* BL21(DE3)	This study

### DNA cloning and plasmid construction of synebrick vectors

BglBrick vectors (e.g., pBbE1k-RFP; Lee et al., 2011) and pSe1Bb1s-GFP (NCBI accession number, KJ814971; Chwa et al., 2016) are standard vectors for construction of SyneBrick vectors. Their four parts are promoter, antibiotic selection marker, gene of interest, and integration site (Table 1). Standard SyneBrick vectors were constructed using circular polymerase extension cloning (CPEC; Quan and Tian, 2009) and conventional restriction-ligation cloning methods with specific primers (Table S1). Using the BglBrick cloning method and a linearized PCR product (eYFP) from p426-TEF-eYFP using eYFP-FW/RV primers, the *gfp* gene was changed to the *eyfp* gene in SyneBrick vectors, yielding pSe1Bb1s-eYFP, and pSe2Bb1k-eYFP from pSe1Bb1s-GFP and pSe2Bb1k-GFP, respectively.

For construction of additional SyneBrick vectors (pSe3 vectors; NSIII), DNA fragments NSIII**a** and NSIII**b** were replaced with NSII**a** and NSII**b** in pSe2Bb1k-GFP and the kanamycin resistance gene was exchanged to chloramphenicol resistance gene. This resulted in pSe3Bb1c-GFP, a gene expression platform in NSIII that was compatible with pSe1Bb1s-GFP and pSe2Bb1k-GFP. For TetR-pTetA based gene expression, pSe1Bb1s-GFP was modified with a functional genetic element of TetR-pTetA from pBbE2c-RFP, yielding pSe1Bb2s-GFP. For T7-based gene expression, pSe2Bb1k-eYFP was modified with a DNA fragment using oligonucleotides containing the T7 sequence (T7-FW/RV), yielding pSe2Bb7k-eYFP. To construct SyneBrick vector 2.0 versions, T7.3-FW and T7.4-FW (containing variant sequences of the original T7 sequences; Temme et al., 2012) were used to generate pSe2Bb7.3k-eYFP and pSe2Bb7.4k-eYFP, respectively.

### Transformation of *S. elongatus* PCC 7942 with synebrick vectors

**Figure 1:**
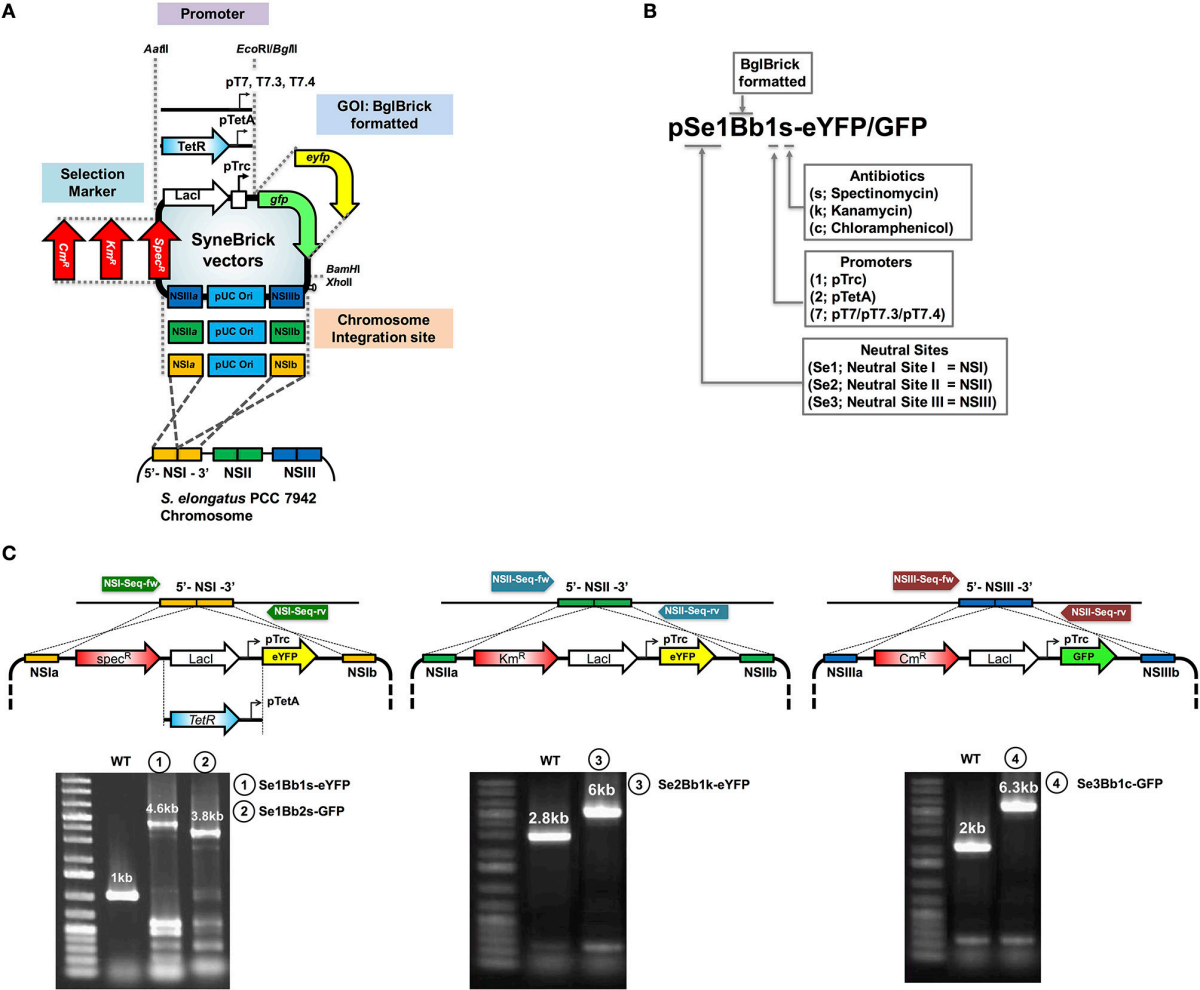
**Design features of SyneBrick vectors for gene expression in ***S. elongatus*** PCC 7942. (A)** SyneBrick vector sections: promoters, antibiotic markers, chromosomal integrational sites and gene of interests (GOI). Standard SyneBrick vector (pSe1Bb1s-GFP) features are two homologous regions of neutral site I (NSIa and NSIb) for chromosomal integration, gene expression of a target under control of the inducible *trc* promoter and LacI repressor, a transcriptional terminator, spectinomycin-resistance selection, and replication of origin (pUC19) for cloning in *E. coli*. Sections are selected to construct a SyneBrick vector. For expansion of SyneBrick vectors, two promoters (TetA and T7 promoters), two antibiotic makers (kanamycin or chloramphenicol) and neutral sites for chromosomal integration (site II or site III) are available as interchangeable parts for the standard SyneBrick vector. **(B)** The nomenclature for the library of SyneBrick vector library is described. SyneBrick vectors constructed in this work are in Table 1. **(C)** Gel images of recombinant cyanobacterial strains. Strains were verified by PCR using oligonucleotides (arrows). Sequences are in Table S1. DNA fragments from wild-type and strains 1, 2, 3, and 4 are shown in gel images. DNA sequences were verified.

Transformation of *S. elongatus* PCC 7942 was as described previously (Golden et al., 1987). Cyanobacterial strains were transformed by incubating at mid-log phase (OD_730_ 1 to 2) with 100 ng plasmid DNA for 24 h in the dark. Cell-DNA mixed cultures were spread on BG-11 plates supplemented with appropriate antibiotics for selecting recombinants (10 μg/mL spectinomycin, 10 μg/mL kanamycin, or 3 μg/mL chloramphenicol). Single colonies were sub-cultured to prevent chromosomal segregation. PCR was used to verify chromosomal integration of targets into either NSI, NSII, or NSIII (Figure 1). DNA sequences were verified using oligonucleotides in Table S1. Genotypes of recombinant *S. elongatus* strains are listed in Table 1.

### Fluorescence measurements of GFP or eYFP in *S. elongatus* PCC 7942

*S. elongatus* mutants with either *gfp* or *eyfp* chromosomally integrated using SyneBrick vectors were cultivated in BG-11 medium overnight at 30°C. To induce GFP or eYFP expression, IPTG for Se1Bb1s-GFP or aTC for Se1Bb2s-GFP was added when cyanobacterial cells were inoculated into BG-11 medium. After 1, 4, or 7 days, 200 μL cell suspensions (diluted to OD_730_ of 1) were transferred to flat bottom, 96-well solid black microtiter plates (Corning, USA) for GFP or eYFP measurements. Fluorescence intensity were measured using an automatic microplate reader (Tecan Infinite M200 pro, Tecan Group Ltd., Switzerland) and optical density was measured by UV-Vis spectrometry. Fluorescence intensities of GFP were measured at excitation/emission wavelengths of 485/535 nm and eYFP was measured at 505/535 nm. Specific fluorescence was calculated by normalizing fluorescence intensity by optical density (730 nm). BG-11 medium was the blank.

## Results and discussion

### SyneBrick vectors: features

To engineer *S. elongatus* PCC 7942 for gene expression, we developed SyneBrick vectors capable of expressing multiple genes controlled by different promoters and transcriptional regulators. An chromosomal integration plasmid with BglBrick cloning features, termed SyneBrick vector, was constructed based on BglBrick (Lee et al., 2011)/CoryneBrick vectors (Kang et al., 2014), using the CPEC cloning method (Quan and Tian, 2011; Figures 1A,B). The set of SyneBrick vectors used three different chromosomal integration sites (Neutral site I, II, and III) where no genetic interference occurred. Although many combinations of neutral sites with antibiotic selection markers (Spec^R^, Km^R^, and Cm^R^) were possible, we chose three pairs of SyneBrick vectors, NSI-Spec^R^, NSII-Km^R^, NSIII-Cm^R^, to generate pSe1Bb1s-GFP, pSe2Bb1k-GFP, and pSe3Bb1c-GFP, respectively. These three vectors can be used for the seven combinatorial construction of the cyanobacterial strains: single integrations, Se1Bb1s, Se2Bb1k, Se3Bb1c; double integrations, Se12Bb11sk, Se13Bb11sc, Se23Bb11kc; and a triple integration, Se123Bb111skc. Additional neutral sites could be identified using transcriptome sequencing analysis (Ng et al., 2015).

The initial SyneBrick vectors have been successfully applied to metabolic engineering (Choi et al., 2016; Chwa et al., 2016; Lee et al., 2017). To expand a selection of the SyneBrick vectors, LacI-pTrc in the standard SyneBrick vectors pSe1Bb1s-GFP and pSe2Bb1k-eYFP were replaced with transcriptional regulator TetR-pTetA and pT7 promoter, respectively. This constructed strains Se1Bb2s-GFP and Se2Bb7k-eYFP. For interpreting the features of SyneBrick vectors, the nomenclature is Se# for *S. elongatus* and neutral sites (#) and Bb for BglBrick vector-originated (Figure 1B). Annotations of BglBrick vectors are one for the *trc* promoter, two for the *tetA* promoter, seven for the *T7* promoter, s for spectinomycin, k for kanamycin, and c for the chloramphenicol resistant gene. Name of genes of interest are followed by a dash.

SyneBrick vectors share the features of BglBrick/CoryneBrick vectors. A PCR step is not necessary for gene cloning and multiple gene assembly is possible with repeated enzyme digestions and ligations without choosing unique restriction enzyme sites. The details of the cloning steps have been described (Lee et al., 2011; Kang et al., 2014). In summary: A target gene of interest digested at *Eco*RI/*Bam*HI sites is ligated to *Eco*RI/*Bgl*II sites of SyneBrick vectors (Table 1) by replacing GFP or eYFP. Each target gene with a BglBrick compatible sequence is sequentially inserted into the SyneBrick vectors in multiple gene clonings. Alternatively, multiple genes built into other BglBrick (Lee et al., 2011) or CoryneBrick vectors (Kang et al., 2014), or BglBrick-formatted genes used in *Synechocystis* sp. PCC 6803 (Huang et al., 2010) or *S. elongatus* PCC 7002 (Markley et al., 2015) can be cloned into SyneBrick vectors by one-step digestion and ligation. Target genes must be checked for the presence of *Eco*RI, *Bgl*II, *Bam*HI, and *Xho*I sites prior to multiple-gene assembly in the vector.

### Development of controllable gene expression for *S. elongatus* PCC 7942

**Figure 2:**
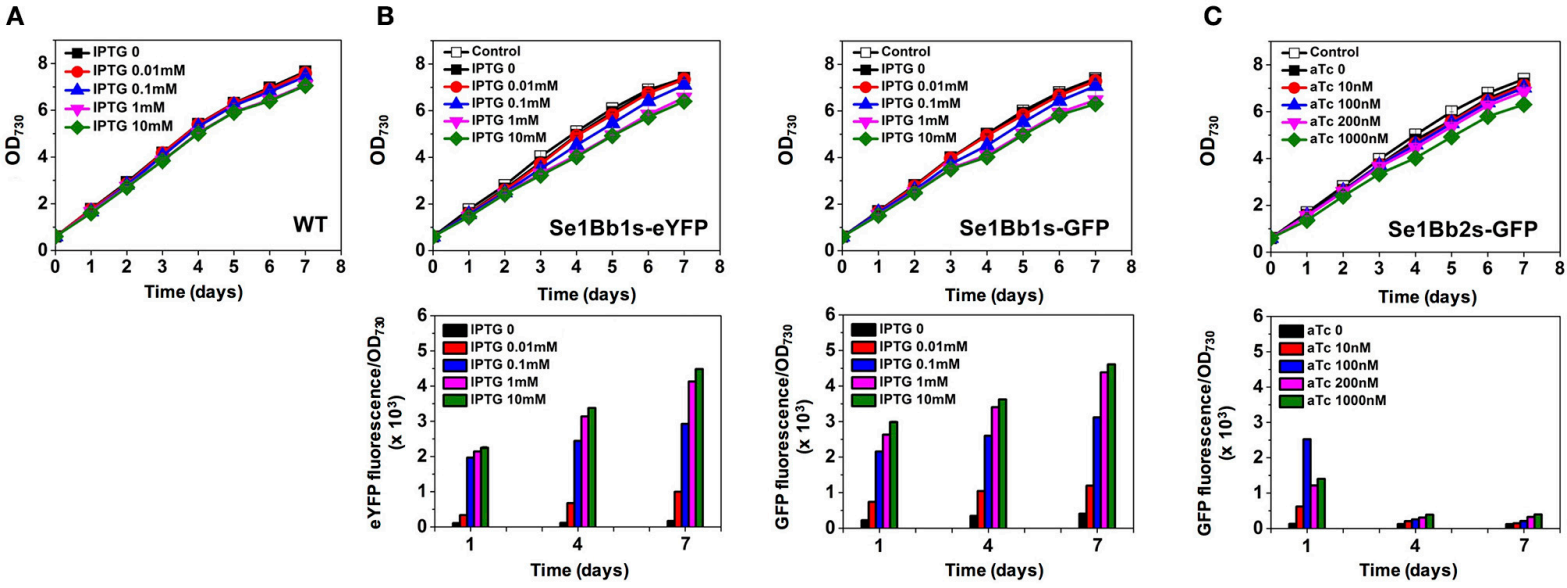
**Gene expression analysis of engineered ***S. elongatus*** PCC 7942 using SyneBrick vectors. (A)** Growth of *S. elongatus* PCC 7942 wild type under 5% (v/v) CO_2_ bubbling in BG-11 supplemented with indicated concentrations of IPTG measured at OD_730_ (black square, 0 mM; red circle, 0.01 mM; blue triangle, 0.1 mM; inverted pink triangle, 1 mM; green diamond, 10 mM). **(B)** Growth of engineered *S. elongatus* PCC 7942 (Se1Bb1s-eYFP and Se1Bb1s-GFP) under 5% (v/v) CO_2_ bubbling in BG-11 supplemented with indicated concentrations of IPTG, measured at OD_730_ (upper panel). Specific fluorescence (intensity per OD_730_) for cyanobacterial cultures (lower panel). Se1Bb1s-None was the control strain (open black square). Symbols for IPTG used are as in **(A)**. **(C)** Strain Se1Bb2s-GFP was used to measure growth and specific fluorescence under aTC induction (black square, 0 mM; red circle, 10 nM; blue triangle, 100 nM; inverted pink triangle, 200 nM; green diamond, 1000 nM). Experiments were performed in triplicate cultures. All data are mean ± standard deviation (*SD*) from triplicate cultures.

To test for controllable gene expression, GFP-expressing, or eYFP-expressing *S. elongatus* PCC 7942 strains (Se1Bb1s-eYFP, Se1Bb1s-GFP, and Se1Bb2s-GFP) were constructed after natural transformation with SyneBrick vectors. We tested the wild-type strain with IPTG up to 10 mM and found no growth inhibition (Figure 2). Subsequently, engineered strains were cultivated in BG-11 medium with 5% (v/v) CO_2_ bubbling in the presence of IPTG or aTc, depending on the strain. GFP or eYFP fluorescent levels were measured.

IPTG induction up to 10 mM and aTc up to 1 mM did not cause growth inhibition of the engineered strains although slight growth inhibition with 10 mM IPTG or 1 mM aTC was observed in the engineered strains compared to the wild type. Strain Se1Bb1s-None was constructed as a control for calibrating values for gene expression of engineered strains because the wild type shows auto-fluorescence in the range of measurements (McEwen et al., 2013). Compared with a control, strains Se1Bb1s-eYFP, Se1Bb1s-GFP, and Se1Bb2s-GFP showed low levels of leaky GFP/eYFP expression in the absence of inducers (Figure 2B). When 0.01, 0.1, 1, and 10 mM of IPTG was used, higher IPTG concentrations resulted in higher induction of the reporter gene. Increased induction levels over cultivation days were also observed. Compared with 0 mM IPTG, induction of Se1Bb1s-GFP with 1 mM resulted in a 10-fold change in gene expression and 10 mM resulted in an 11-fold change. This result was consistent with the 16-fold induction of β-glucuronidase activity in a reporter assay using an IPTG-inducible pTrc gene expression system in *S. elongatus* PCC 7942 (Geerts et al., 1995). Compared with Se1Bb1s-GFP, Se1Bb1s-eYFP had a 24-fold higher induction with 1 mM IPTG and 26-fold with 10 mM. This discrepancy could be due to differences in signal intensities between intracellular GFP and eYFP in cyanobacteria. At least 10-fold induction was achieved with either 1 or 10 mM IPTG in *S. elongatus* PCC 7942 using SyneBrick vectors. For controllable gene expression with SyneBrick vectors with the 1 promoter, 1 mM IPTG induction is recommended.

Se1Bb2s-GFP with the TetR-pTetA transcriptional system was tested with four concentrations of aTc: 10, 100, 200, and 1000 nM in 5% (v/v) CO_2_. The highest induction of 19-fold occurred when 100 nM aTc was used, compared to without aTc (Figure 2C). However, induction was decreased dramatically when more than 100 nM aTC was used. Thus, 100-nM aTC induction was recommended with SyneBrick vectors with the 2 promoter and 24-h culture. In previous studies, an aTc-dependent induction system for *Synechococcus* sp. PCC 7002 had induction ranges of 6- and 32-fold with 1000 ng/mL (≅ 2200 nM) aTc (Zess et al., 2016) until 48 h. Dynamic ranges of inductions up to 290-fold have been reported for *Synechocystis* sp. PCC 6803 under light-activated heterotrophic growth with 10 μg/mL (≅ 22,000 nM) aTC for 48 h (Huang and Lindblad, 2013). Consistent with these results, our TetR system showed decreased gene expression after 48 h due to aTc degradation in light. However, our TetR-pTetA systems required much less aTC to induce gene expression, compared with previous studies (Huang and Lindblad, 2013; Zess et al., 2016). Thus, time-dependent gene expression in Se1Bb2s-GFP under light conditions could be possible by adding aTc in a pulse-type control mode. Engineering of our TetR-pTetA transcriptional system in *S. elongatus* PCC 7942 with light wavelength (i.e., red light or white light) and intensities carefully considered for SyneBrick applications with aTC as a light-sensitive inducer. Sequence modification of the pTetA promoter with tetO operators could alter intrinsic expression and induction of target genes. This could be easily incorporated into SyneBrick platform vectors.

### Expanding synthetic biology platforms using T7 gene expression systems

**Figure 3:**
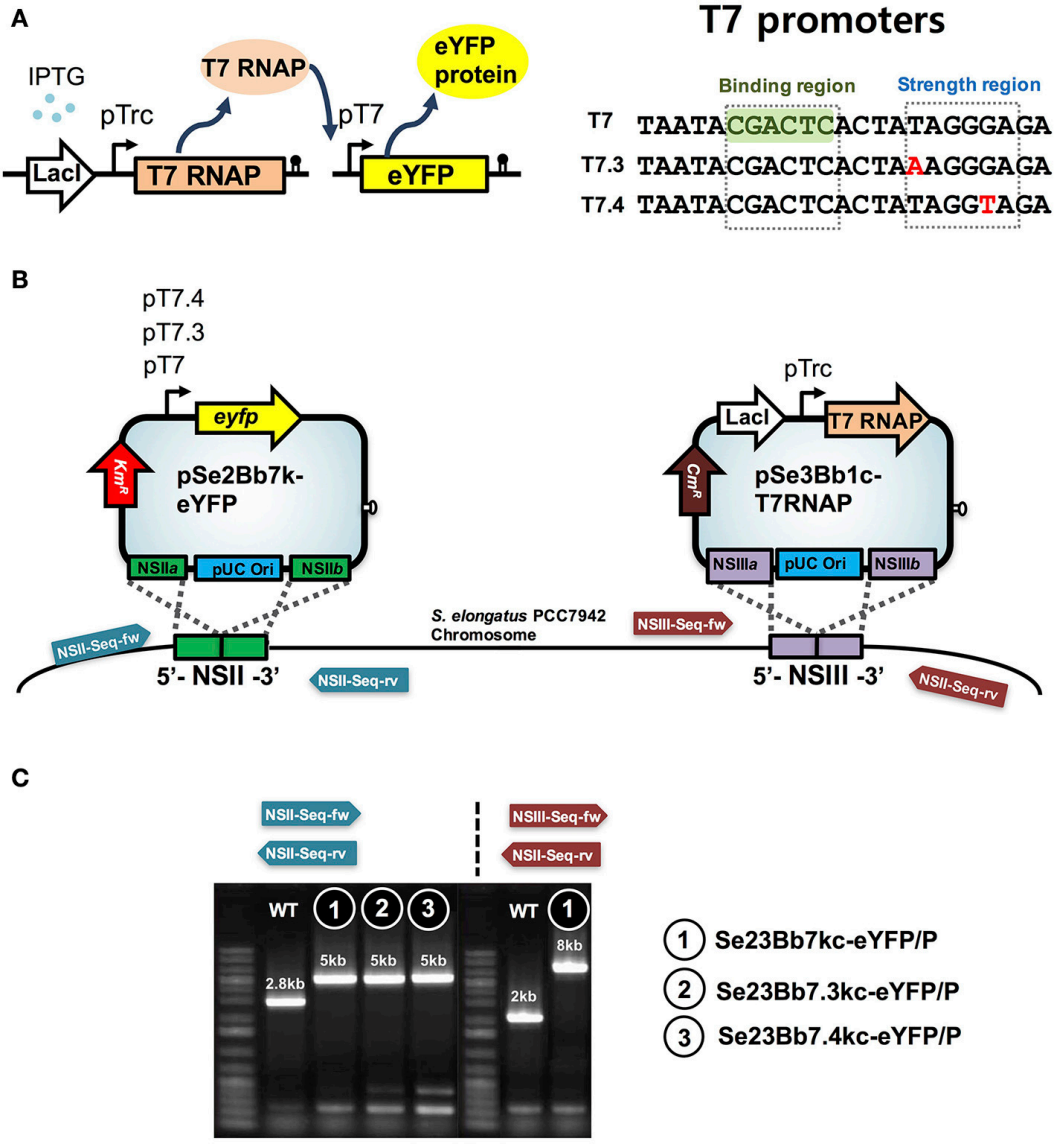
**Design features of SyneBrick vectors 2.0 for gene expression using the T7 promoter in ***S. elongatus*** PCC 7942. (A)** Scheme for genetic control of target gene (e.g. eYFP). With IPTG, T7 RNA polymerase (T7 TNAP) was expressed and its cognate T7 promoter was used to express target genes. Sequences of original T7 promoter and sequence variants (T7.3 and T7.4) with binding and strength regions (Temme et al., 2012). **(B)** Construction of cyanobacterial strains using pSe2Bb7k-eYFP, pSe2Bb7.3k-eYFP, pSe2Bb7.4k-eYFP, and pSe3Bb1c-T7RNAP vectors for expression of eYFP protein under control of T7 variant promoters. Sequencing primers are in Table S1. **(C)** Gel images of recombinant cyanobacterial strains. Strains were verified by PCR using oligonucleotides (arrows). Sequences are in Table S1. DNA fragments from wild-type and strains 1, 2, and 3 are shown in gel images. DNA sequences were verified.

To narrow the dynamic range of IPTG induction with tunable gene expression in *S. elongatus* PCC 7942, T7 RNA polymerase (RNAP) and its cognate T7 promoter were developed in SyneBrick vectors (pSe2Bb7k-eYFP and pSe3Bb1c-T7RNP; Figure 3). We tested the possibility of T7 promoter-driven transcription in the absence of T7 RNAP. No gene expression was observed in Se2Bb7k-eYFP under control of the T7 promoter, indicating that the T7 promoter was not recognized by cyanobacterial RNA polymerases (data not shown). Thus, the T7 RNAP *gene 1* encoding for T7 RNAP from *E. coli* BL21(DE3) was integrated into Se2Bb7k-eYFP using pSe3Bb1c-T7RNP, yielding strain Se23Bb7kc-eYFP/P.

**Figure 4:**
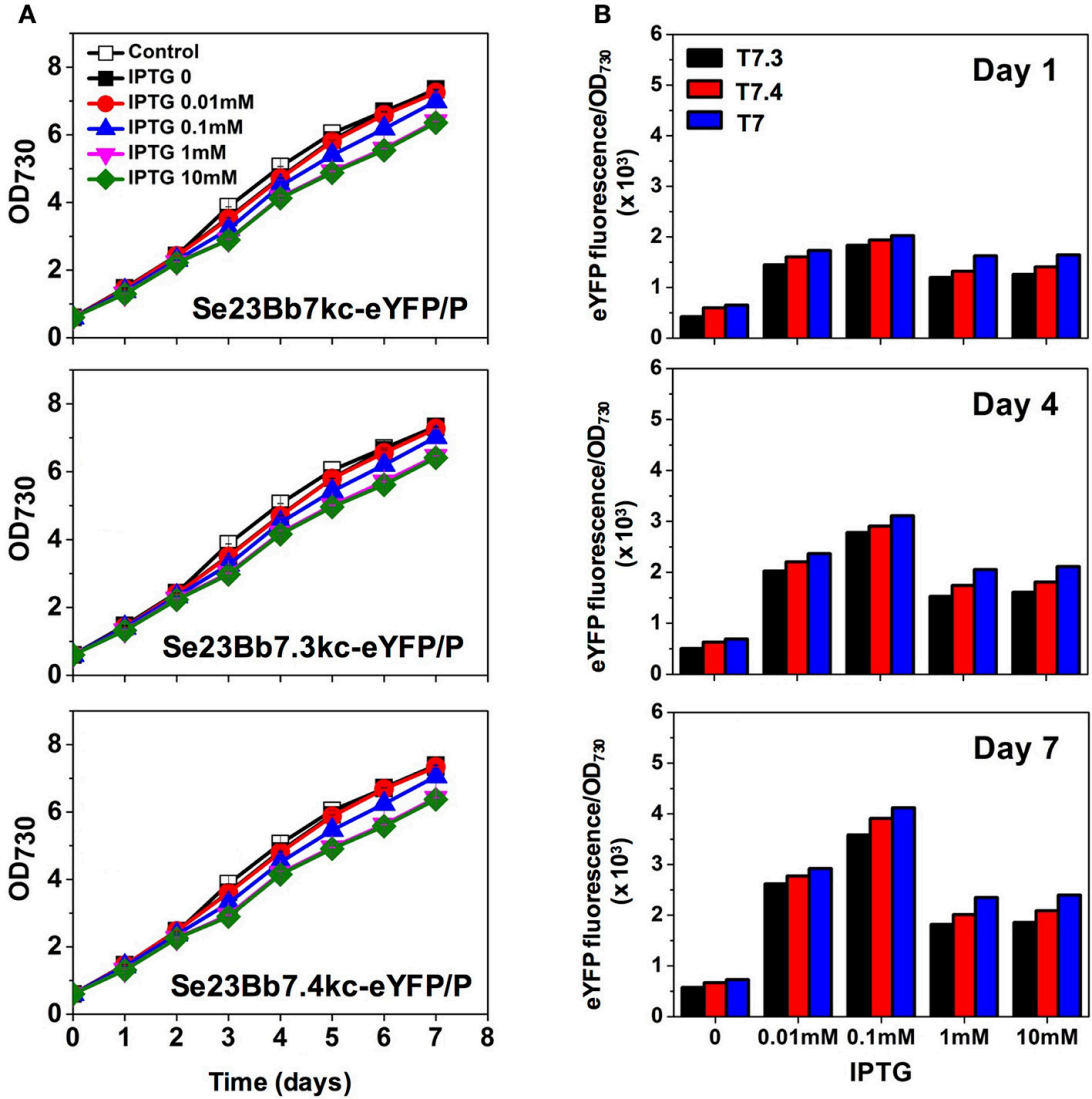
**Gene expression analysis of engineered ***S. elongatus*** PCC 7942 using SyneBrick 2.0 vectors. (A)** Growth of engineered *S. elongatus* PCC (pSe23Bb7kc-eYFP/P, pSe23Bb7.3kc-eYFP/P, and pSe23Bb7.4kc-eYFP/P) under 5% (v/v) CO_2_ bubbling in BG-11 supplemented with indicated concentrations of IPTG, measured at OD_730_ (black square, 0 mM; red circle, 0.01 mM; blue triangle, 0.1 mM; inverted pink triangle, 1 mM; green diamond, 10 mM). **(B)** Specific fluorescence (intensities per OD_730_) were calculated for strains. Experiments were performed in triplicate cultures. All data are presented as mean ± standard deviation (*SD*) from triplicate cultures.

Compared with the LacI-pTrc gene expression system of Se1Bb1s-eYFP, the T7RNAP-T7 gene expression system of Se23Bb7kc-eYFP/P showed efficient induction of target gene with IPTG. Specific fluorescence intensities of Se23Bb7kc-eYFP/P increased 6-fold with 0.01 mM IPTG and 1.5-fold with 0.1 mM compared to Se1Bb1s-eYFP (Figure 4). Thus, induction of the T7RNAP-T7 gene expression system with 0.1 mM IPTG resulted in the same levels of induction as 1 mM IPTG in the LacI-pTrc gene expression system. The efficient induction with the low IPTG concentration could be due to an inherently fast mRNA elongation rate of the T7 RNA polymerase. Decreased gene expression was detected when more than 1 mM IPTG was used. The reason that expression was reduced with high concentrations of IPTG was because the RNA polyadenylation and degradation mechanisms in cyanobacteria are different from *E. coli* (Rott et al., 2003). Thus, orthogonal expression of the T7 polymerase *gene 1* can be alternatively applied by replacing the pTrc promoter with the pTetA promoter to prevent interference with expression from the T7 promoter. The T7RNAP-T7 gene expression system induced well with 0.01 or 0.1 mM IPTG.

In addition, variants of T7 promoters (T7.3 and T7.4) in which a 5-bp strength-determining region was modified (Temme et al., 2012) were replaced with the original T7 promoter to alter promoter strength without RNA specificity (Figure 3). This generated strain Se23Bb7.3kc-eYFP/P with 82% eYFP expression compared to Se23Bb7kc-eYFP/P and Se23Bb7.4kc-eYFP/P, with 90% expression. These reductions in gene expression in cyanobacteria are different from gene expression levels in *E. coli*, which are 28% for T7.3 and 17% for T7.4 from T7 (Temme et al., 2012). The order of promoter strength using a T7 library in cyanobacteria was consistent with *E. coli*. Using a library of T7 promoter SyneBrick vectors, the process of refactoring of a gene cluster encoding for complex proteins such as α-carboxysomes would be possible as rewriting the genomes to decipher their functions in cyanobacteria. T7 lysozyme can also be used as an inhibitor for fine-tuning gene expression of the T7 promoter by controlling T7 RNA polymerase activity.

The development of clustered regulatory interspaced short palindromic repeats (CRISPR) interference technology has allowed control of gene expression by inducing dCas9 encoding genes with aTc. This method has successfully been applied to lactate production from 1% CO_2_ (Gordon et al., 2016) and to repress multiple genes in the cyanobacterial genome (Yao et al., 2016). Thus, SyneBrick vectors could be further expanded to precisely control gene expression by integrating CRISPR technology for metabolic engineering of *S. elongatus* PCC 7942.

## Conclusion

A synthetic biology platform of SyneBrick vectors for gene expression in *S. elongatus* PCC 7942 was developed with different gene inducible systems and chromosomal integration sites. LacI-pTrc, TetR-pTetA, T7RAP-T7-based SyneBrick vectors controlled expression of target genes and showed strong gene expression in *S. elongatus* PCC 7942 under 5% CO_2_. A synthetic T7 promoter library was used for a broad range of gene expression in *S. elongatus* PCC 7942. Thus, the SyneBrick vectors could be useful for expressing target genes in synthetic biology to engineer *S. elongatus* PCC 7942 as a biosolar cell factory for directly converting CO_2_ to value-added chemicals.

## Author contributions

WK and HW designed the research; WK performed the research; WK, SL, YU, SS, and HW analyzed the data; WK and HW drafted the manuscript; WK, SL, YU, SS, and HW contributed to the final manuscript.

## Funding

This work was financially supported by Korea CCS R&D Center (KCRC) (no. 2014M1A8A1049277) and the National Research Foundation of Korea grant-funded by the Korean Government (Ministry of Science, ICT & Future Planning) (2016, University-Institute Cooperation program). Also, this work is partially supported by Golden Seed Project (213008-05-1-WT911) grant, funded by Ministry of Agriculture, Ministry of Oceans and Fisheries.

### Conflict of interest statement

The authors declare that the research was conducted in the absence of any commercial or financial relationships that could be construed as a potential conflict of interest.
