# Supplementary material for: Development of SyneBrick Vectors As a Synthetic Biology Platform for Gene Expression in Synechococcus elongatus PCC 7942
Source: Front Plant Sci. 2017 Mar 2;8:293. doi: 10.3389/fpls.2017.00293 (PMC5332412; doi:10.3389/fpls.2017.00293)
Supplement: Supplementary file 1 [file Table1.DOCX]

Table S1. Oligonucleotides used in this work

| Oligonucleotides | Sequences (5’-3’) | | Restriction enzyme | |
| --- | --- | --- | --- | --- |
| **Oligonucleotides for construction of SyneBrick vectors** | | | | |
| NSI-seq-FW | | ACA TCT TCC TGC TCC AGA AG | |  |
| NSI-seq-RV | | AGC AGG TCG TTC AAA GAC TG | |  |
| NSII-seq-FW | | GGC TAC GGT TCG TAA TGC CA | |  |
| NSII-seq-RV | | GAG ATC AGG GCT GTA CTT AC | |  |
| NSIII-seq-FW | | GTC ACC GTG ACG GTA CCC AG | |  |
| NSIII-seq-RV | | CGA AGC TGC TGC TTG AGA AC | |  |
| NSIII-a-FW | | CTC ACA TGT GTG CTG GGC CCT CAG CCA GCT CGT CGT GAT G | |  |
| NSIII-a-RV | | GAG CTC TCT AGA GAC CGA TCA ACC AGT CCC TC | |  |
| NSIII-a-vec-FW | | GAG GGA CTG GTT GAT CGG TCT CTA GAG AGC TC | |  |
| NSIII-a-vec-RV | | CAT CAC GAC GAG CTG GCT GAG GGC CCA GCA CAC ATG TGA G | |  |
| NSIII-b-FW | | CAC TCA AAG GCG GTA ATA CGG ACA AGC CGG GGC AGA CGT G | |  |
| NSIII-b-RV | | ATT TTG GTC ATG AGA TTA TCA CAG TCG GCG TCA CGG CAA C | |  |
| NSIII-b-vec-FW | | GTT GCC GTG ACG CCG ACT GTG ATA ATC TCA TGA CCA AAA T | |  |
| NSIII-b-vec-RV | | CAC GTC TGC CCC GGC TTG TCC GTA TTA CCG CCT TTG AGT G | |  |
| Cm-vec-FW | | CCA GTG ATT TTT TTC TCC ATT TTA GCT TCC TTA GCT CCT G | |  |
| Cm-vec-RV | | GAT GAG TGG CAG GGC GGG GCG TAA TTT GAT ATC GAG CTC TC | |  |
| Cm-FW | | GAG AGC TCG ATA TCA AAT TAC GCC CCG CCC TGC CAC TCA TC | |  |
| Cm-RV | | CAG GAG CTA AGG AAG CTA AAA TGG AGA AAA AAA TCA CTG G | |  |
| T7-FW | | TTT **GAC GTC** TAA TAC GAC TCA CTA TAG GGA ATT CAA AAG ATC TTT TAA G | | *Aat*II |
| T7.3-FW | | TTT GAC **GTC GAC** TAA TAC GAC TCA CTA AAG GGA GAG AAT TCA AAA GAT CTT TTA AGA AGG AG | | *Sal*I |
| T7.4-FW | | TTT GAC **GTC GAC** TAA TAC GAC TCA CTA TAG GTA GAG AAT TCA AAA GAT CTT TTA AGA AGG AG | | *Sal*I |
| T7-RV | | TTT **GGA TCC** TTA TTT GTA GAG CTC ATC CAT GCC ATG TG | | *Bam*HI |
| T7RNAP-FW | | TTT **GAA TTC** AAA **AGA TCT** TCC ACA CTA ACT CCA AAG GCA GGC AAA TTA TGA ACA CGA TTA ACA TCG C | | *Eco*RI/*Bgl*II |
| T7RNAP-RV | | TTT **GGA TCC** TTA CGC GAA CGC GAA GTC CG | | *Bam*HI |
| eYFP-FW | | TTT CA**G** **AAT** **TC**A AA**A GAT CT**G GGA GGG TCA AAG  AGC GAA GGAGGT TAA TCA TGT CTA AAG GTG AAG AAT TAT TCA CTG G | | *Eco*RI/*Bgl*II |
| eYFP-RV | | TTT **GGA TCC** TTA TTT GTA CAA TTC ATC CAT ACC ATG GG | | *Bam*HI |

Note: Restriction enzyme sequences were bolded.
